# Blockade of Intranigral and Systemic D3 Receptors Stimulates Motor Activity in the Rat Promoting a Reciprocal Interaction among Glutamate, Dopamine, and GABA

**DOI:** 10.3390/biom9100511

**Published:** 2019-09-20

**Authors:** Marina Rodríguez-Sánchez, Rodrigo Erick Escartín-Pérez, Gerardo Leyva-Gómez, José Arturo Avalos-Fuentes, Francisco Javier Paz-Bermúdez, Santiago Iván Loya-López, Jorge Aceves, David Erlij, Hernán Cortés, Benjamín Florán

**Affiliations:** 1 Departamento de Farmacología, Centro de Investigación y de Estudios Avanzados del Instituto Politécnico Nacional, Ciudad de México 07360, Mexico; 2 Laboratory of Neurobiology of Eating, Universidad Nacional Autónoma de México, FES Iztacala, Ciudad de México 54090, Mexico; 3 Departamento de Farmacia, Facultad de Química, Universidad Nacional Autónoma de México, Ciudad de México 04510, Mexico; 4 Departamento de Fisiología, Biofísica y Neurociencias, Centro de Investigación y de Estudios Avanzados del Instituto Politécnico Nacional, Ciudad de México 07360, Mexico; 5 Department of Physiology SUNY Downstate Medical Center, Brooklyn, NY 11203, USA; 6 Laboratorio de Medicina Genómica, Departamento de Genética, Instituto Nacional de Rehabilitación Luis Guillermo Ibarra Ibarra, Ciudad de México 14389, Mexico

**Keywords:** D3 receptors, subthalamo–nigral pathway, substantia nigra reticulata, D1-like receptors

## Abstract

In vivo activation of dopamine D3 receptors (D3Rs) depresses motor activity. D3Rs are widely expressed in subthalamic, striatal, and dendritic dopaminergic inputs into the substantia nigra pars reticulata (SNr). In vitro studies showed that nigral D3Rs modulate their neurotransmitter release; thus, it could be that these changes in neurotransmitter levels modify the discharge of nigro-thalamic neurons and, therefore, motor behavior. To determine how the in vitro responses correspond to the in vivo responses, we examined the effect of intra-nigral and systemic blockade of D3Rs in the interstitial content of glutamate, dopamine, and GABA within the SNr using microdialysis coupled to motor activity determinations in freely moving rats. Intranigral unilateral blockade of D3R with GR 103,691 increased glutamate, dopamine, and GABA. Increments correlated with increased ambulatory distance, non-ambulatory activity, and induced contralateral turning. Concomitant blockade of D3R with D1R by perfusion of SCH 23390 reduced the increase of glutamate; prevented the increment of GABA, but not of dopamine; and abolished behavioral effects. Glutamate stimulates dopamine release by NMDA receptors, while blockade with kynurenic acid prevented the increase in dopamine and, in turn, of GABA and glutamate. Finally, systemic administration of D3R selective antagonist U 99194A increased glutamate, dopamine, and GABA in SNr and stimulated motor activity. Blockade of intra-nigral D1R with SCH 23390 prior to systemic U 99194A diminished increases in neurotransmitter levels and locomotor activity. These data highlight the pivotal role of presynaptic nigral D3 and D1R in the control of motor activity and help to explain part of the effects of the in vivo administration of D3R agents.

## 1. Introduction

Activation of dopamine D3 receptors (D3Rs) depresses motor activity in rodents, as pharmacologically selective antagonists increase spontaneous motor behavior, whereas agonists decrease it [1,2,3,4,5,6,7,8,9,10,11,12,13,14,15]. Messenger RNA (mRNA), protein, and in vitro neurochemical and electrophysiological studies indicate that D3Rs are widely expressed in projections into the substantia nigra pars reticulata (SNr) of the rat, particularly in striato–nigral [16,17,18,19,20] and subthalamo–nigral projections [21,22,23,24,25,26]. In addition, dopaminergic neurons that release dopamine from their dendrites within the SNr also express D3R [21,27,28,29,30,31,32]. The functional effects of these presynaptic D3Rs modulating neurotransmitter release have also been described elsewhere [19,20,23,25,26]. Because discharges of nigro-thalamic neurons control the flow of impulses through the thalamic relay nucleus to the motor cortex [33], the activation of D3R in the SNr may play a major role in the modulation of motor behavior. Therefore, to determine how the in vitro effects correspond with the in vivo responses and how the D3R effects in the different intra-nigral afferents modify neurotransmitter release, we have examined the responses to blockading D3R activity by systemic and intra-nigral antagonist administration using microdialysis and motor activity determinations in freely moving animals. Previous information on these data appears in a meeting report [34].

## 2. Materials and Methods

### 2.1. Animals

Male Wistar rats of 200–240 g, housed together (five per cage) with water and food available ad libitum and kept under natural light cycle, were used throughout. All the procedures were conducted in accordance with the National Institute of Health Guide for Care and Use of Laboratory Animals and were approved by the Institutional Animal Care Committee of the CINVESTAV, taking all efforts to minimize suffering and the number of animals used. The number of animals used in this study was 44. 

### 2.2. Microdialysis Coupled to Behavioral Measurements

Rats were anesthetized with ketamine/xylazine (112.5/22.5 mg/kg i.p.), and stereotaxically implanted with a CMA/11 guide cannula at coordinates −8.7 lateral, −1.9 dorso-ventral, and −7.0 mm from dura with an inclination of 43 degrees with respect to interaural line to access the right SNr [35]. To prevent lumen occlusion, a stainless steel stylet was inserted into the guide cannula. Rats were allowed full recovery until reaching their pre-surgical body weight (3–4 days) before the beginning of the microdialysis experiments. 

Microdialysis probes with a 1 mm membrane (CMA/11, O.D. 0.24 mm, polycarbonate/polyether copolymer dialysis membrane, and molecular weight cut-off 6000 Daltons) were used. In vitro recovery of the microdialysis probes was 9.1 ± 1.4% for dopamine, 7.8 ± 0.9% for gamma-aminobutyric acid (GABA), and 10.7 ± 1.8% for glutamate at a flow rate of 3 µL/min using a CMA/100 perfusing pump. The composition of the perfusing solution was, in mM, as follows: NaCl 137, KCl 3, CaCl_2_ 1.2, MgSO_4_ 1, NaH_2_PO_4_ 2, and Dextrose 3, buffered to pH 7.4. For experiments, the stylet inserted into the microdialysis guide cannula was carefully replaced with the probe, which was connected to the perfusion system. Rats were then subjected with an abdominal belt that did not interfere with free movement and were placed into an infrared photobeam-controlled open-field activity test arena: 43.38 × 43.38 × 30.28 cm (Activity Test Chamber ENV-515s, MED Associates Inc., St Albans, VT, USA). Motor activity was evaluated during the experiment and locomotor parameters were automated recorded by the software provided by manufacturer. The perfusion system was stabilized by at least 2 h and then samples of 60 µL were collected every 20 min using a refrigerated fraction collector and immediately analyzed with high pressure liquid chromatography (HPLC) systems coupled to electrochemical (GABA and glutamate) or fluorescence detection (dopamine). Ten to thirteen fractions were collected throughout the experiment. 

The separation of GABA and glutamate was achieved in a C18 column (particle size 2.7 µm, 4.6 mm width, and 100 mm long; SUPELCO Analytical (Sigma-Aldrich Co, St. Louis, MO, USA) with a mobile phase of 100 mM disodium hydrogen phosphate, 30% methanol, 3.5% acetonitrile, and pH 6.7 adjusted with phosphoric acid 70% at 32.5 °C. Both neurotransmitters were measured by precolumn derivatization with o-phthalaldehyde (OPA) [36,37]. Briefly, derivation was achieved by mixing 5 µL of working derivation solution (2.7 mg OPA, 10% methanol, 0.5 µL 2-β-mercaptoethanol (99%), and 90% 0.1 M sodium tetraborate buffer) with 35 µL of filtered microdialysis samples (nylon-membrane/0.45 lm pore size) and detected with a glassy carbon electrode VT-03 (Antec Leyden, Zoeterwoude, Netherlands) set at −550 mV (with respect to an Ag/AgCl reference electrode) [37]. For DA separation, 25 µL of microdialysis samples was injected into a C18 column (particle size 2.7 µm, 4.6 mm width, and 100 mm long; SUPELCO Analytical from Sigma-Aldrich Co (St. Louis, MO, USA)) with mobile phase (monocloroacetic acid 100, EDTA 0.507, 1-octanosulfonic acid 0.767 (in mM), and acetonitrile 4.5%, pH NaOH adjusted to 3.2), coupled to a Waters 2475 Multi λ Fluorescence Detector (Waters Corporation, Milford, MA, USA), λx 279 nM, and λe 320 nM [36]. With this method, basal concentration of neurotransmitters measured in dialysates from SNr were, in fmol/µL, as follows: dopamine 5.82 ± 1.09, GABA 33.54 ± 3, and glutamate 298 ± 28 (*n* = 44 animals)

**Figure 1:**
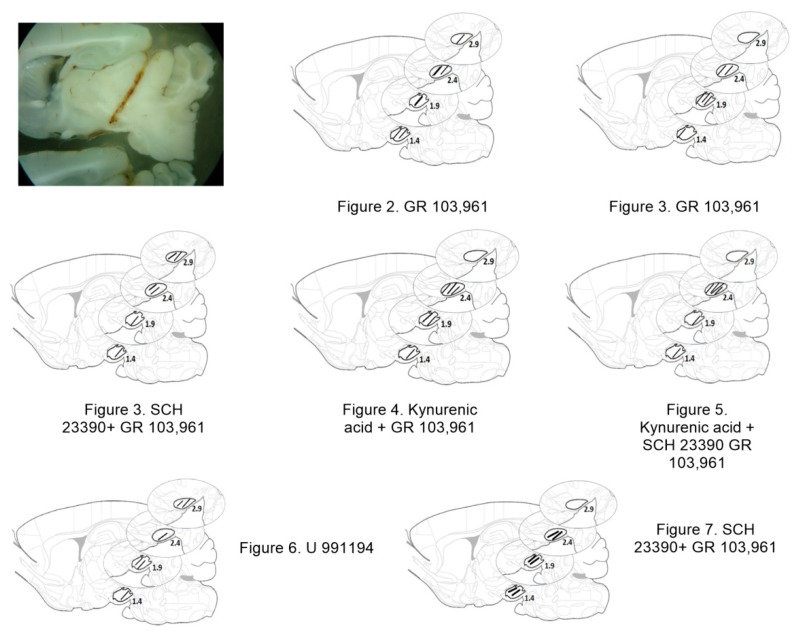
Localization of the tip of the microdialysis cannula within substantia nigra pars reticulata (SNr) of the experiments of this project. At the upper left is shown a microphotograph of a sagittal slice of the brain of a rat, showing the trajectory of guide cannula and the tip of the dialysis cannula within the SNr. Images like this were obtained and compared with schematic diagrams from the Atlas of Paxinos and Watson [38], the location of the tip of the cannula in the different experiments of this project is indicated with the number of the corresponding figure.

At the end of the experiments, rats were deeply anesthetized with a mixture of ketamine/xylazine (152.5/32.5 mg/kg i.p.) and decapitated. Brains were removed and post-fixed in 4% formaldehyde solution during 24 h, and then they were sectioned at 300 μm in the sagittal plane, photographed, and compared with sections of the atlas of Paxinos and Watson [38] in order to determine the adequate location of the cannula. Figure 1 shows a representative sagittal section of the brain of one rat, showing the location of the probe and schematic drawings of the probe locations according to the atlas of Paxinos and Watson [38]. Black lines represent the position of the dialysis membrane in a representative plane of the SNr (from back to front: 1.4 mm, 1.9 mm, 2.4 mm, and 2.9 mm lateral to bregma); the number of the figure of the set of experiment that corresponds to each animal and the treatment administered is also indicated.

### 2.3. Experimental Design and Statistical Analysis

**Figure 2:**
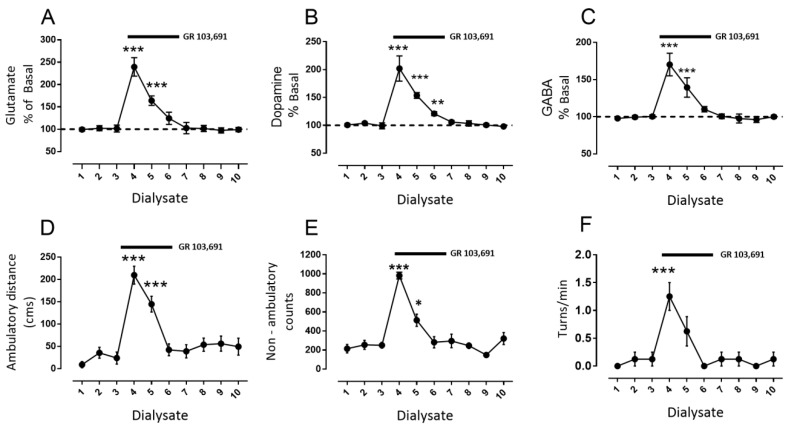
Intra-nigral dopamine D3 receptor (D3R) blockade increases interstitial glutamate, dopamine, and GABA and stimulates locomotor activity. (**A**) shows the percentage of change of interstitial nigral glutamate in dialysate X the respect to dialysate 1 (baseline); the black bar indicates the period of perfusion of D3R selective antagonist GR 103,691 (100 nM). (**B**,**C)** are the same as (**A**), but with dopamine and GABA, respectively. In (**D**–**F**), the change is depicted in ambulatory distance in cm, the non-ambulatory activity in light-beam counts and circling in turns/min, respectively, in the 20 min period during which the respective dialysate was collected. * *p* < 0.05, ** *p* < 0.001, and *** *p* < 0.001 with respect to dialysate three (before antagonist perfusion); two-way ANOVA followed by Tukey, *n* = 8 rats.

**Figure 3:**
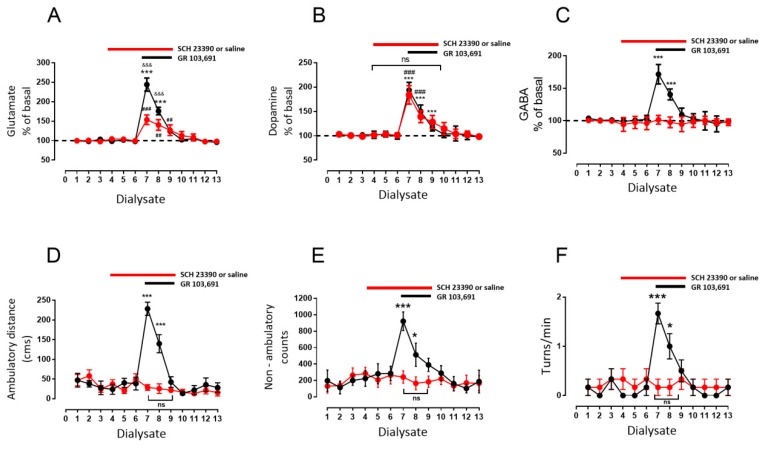
Blockade of intra-nigral D1R prevents the behavioral effect of intra-nigral D3R antagonist and decreases the increments of glutamate and GABA. (**A**–**C**) demonstrate the effect of intra-nigral D1R blockade with SCH 23,390 (1 µM, from dialysate three to eight) on the interstitial concentration of glutamate, dopamine, and GABA, alone or in co-perfusion, of D3R antagonist GR 103,691 (100 nM, from dialysate six to eight). Black circles indicate a group of animals perfused with saline instead of SCH 23,390 as control for the red-circles group, perfused with the drug prior to the D3R blockade. (**D**–**F**) exhibit the behavioral effects of co-superfusion of the antagonists in ambulatory distance, non-ambulatory counts, and in turns/min, respectively. * *p* < 0.05 and *** *p* < 0.001 with respect to dialysate six (black-circles group); ### *p* < 0.001 with respect to dialysate six (red-circles group); and &&& *p* < 0.001 (black-circles group with respect to red circles); ns, without significant differences between dialysates. Two-way ANOVA followed by Tukey, *n* = 6 rats per group.

**Figure 4:**
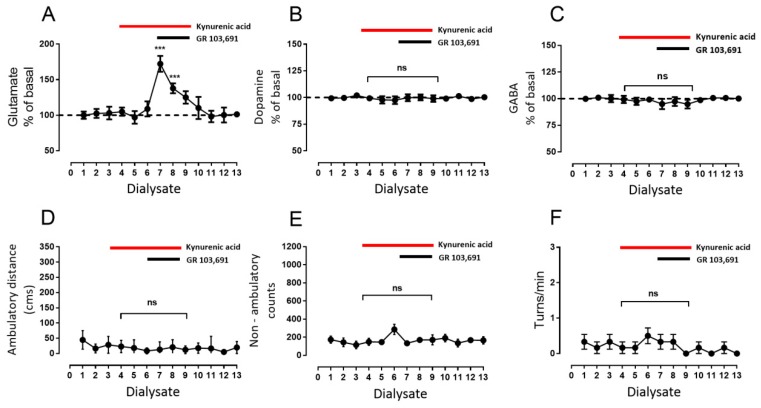
Blockade of intra-nigral NMDA receptor (NMDAR) prevents increments of dopamine, GABA, and behavioral effects, but not of glutamate, induced by D3R blockade. (**A**–**C)** show the effect of intra-nigral NMDAR blockade with kynurenic acid (500 µM, from dialysate three to eight) on the interstitial concentration of glutamate, dopamine, and GABA, alone or in co-perfusion, of D3R antagonist GR 103,691 (100 nM, from dialysate six to eight). (**D**–**F)** present the behavioral effects of co-superfusion of the antagonists in ambulatory distance, non-ambulatory counts, and turns/min, respectively. *** *p* < 0.001 with respect to dialysate six; ns, non-significant differences between dialysates. Two-way ANOVA followed by Tukey, *n* = 6 rats.

**Figure 5:**
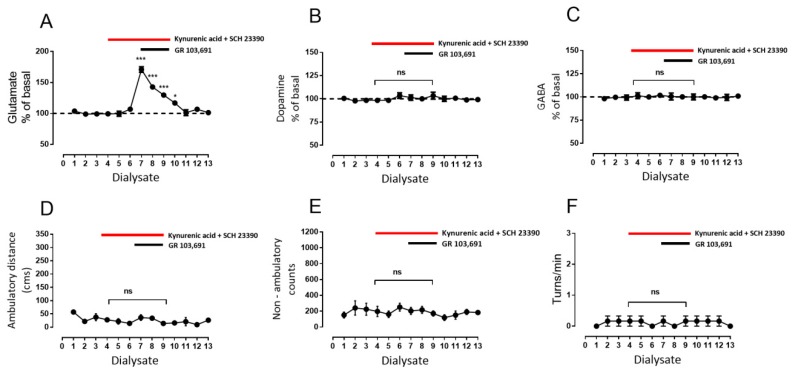
Concomitant blockade of NMDAR and D1R prevents increments of dopamine, GABA, and behavioral effects, but not of glutamate, induced by D3R blockade. (**A**–**C**) illustrate the effect of intra-nigral NMDAR blockade with kynurenic acid and D1R with SCH 23,390 (500 µM and 1 µM, respectively from dialysate three to eight) on interstitial concentration of glutamate, dopamine, and GABA, together or in co-perfusion, of D3R antagonist GR 103,691 (100 nM, from dialysate six to eight). (**D**–**F**) present the behavioral effects of co-superfusion of the antagonists in ambulatory distance, non-ambulatory counts, and turns/min, respectively. * *p* < 0.05 and *** *p* < 0.005 with respect to dialysate six; ns, non-significant differences between dialysates. Two-way ANOVA followed by Tukey, *n* = 6 rats.

**Figure 6:**
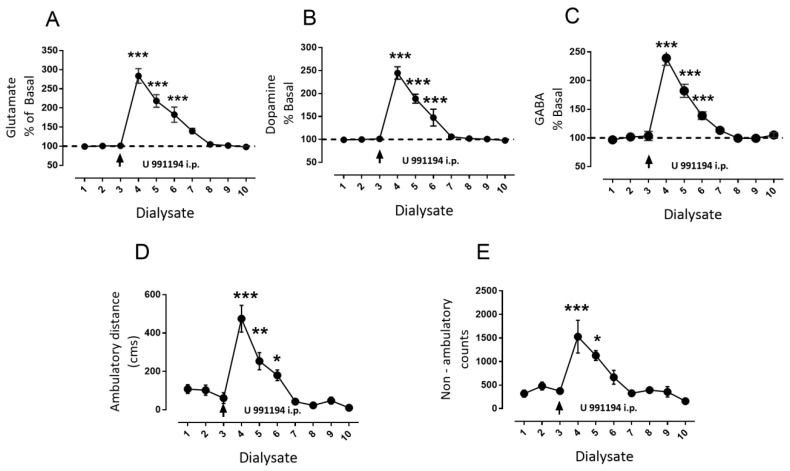
Systemic blockade of D3R with U 99194A also increased intra-nigral glutamate, GABA, dopamine, and locomotor activity. (**A**) reveals the percentage of change of interstitial nigral glutamate in dialysate X with respect to dialysate 1 (baseline), and the arrow indicates the injection of a single dose of D3R selective antagonist U 99194A (25 mg/kg i.p.). (**B**,**C**) are the same as (**A**), but with dopamine and GABA, respectively. In (**D**,**E**), change in ambulatory distance is in cm, and non-ambulatory activity is in light-beam counts in the 20 min period during which the respective dialysate was collected. * *p* < 0.05, ** *p* < 0.001, and *** *p* < 0.001 with respect to dialysate three (before antagonist injection), two-way ANOVA followed by Tukey, *n* = 6 rats.

**Figure 7:**
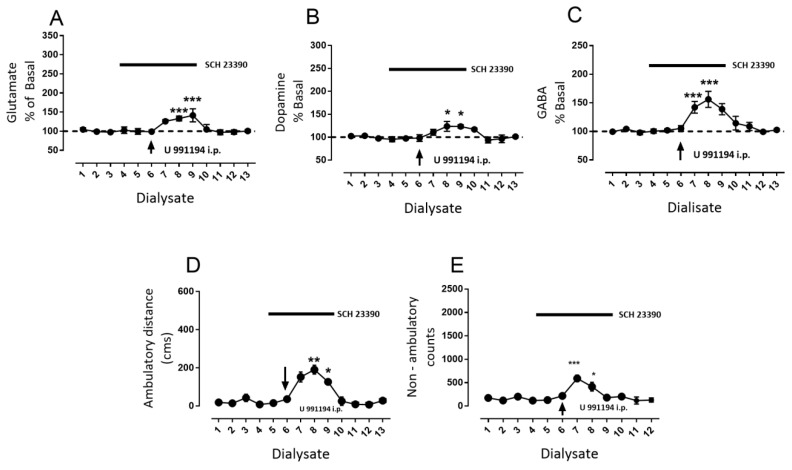
Intra-nigral blockade of D1R diminishes increments in the neurotransmitter and locomotor activity elicited by D3R systemic blockade. (**A**) the percentage is shown of the change of interstitial nigral glutamate in dialysate X with respect to dialysate 1 (baseline), with the bar indicating the intra-nigral perfusion of D1R antagonist SCH 23,390 (1 µM), and the arrow indicating the injection of a single dose of D3R selective antagonist U 99194A (25 mg/kg i.p.). (**B**,**C**) are the same as (**A**), but with dopamine and GABA, respectively. In (**D**,**E**), the change is shown in ambulatory distance in cm, and non-ambulatory activity in light-beam counts in the 20 min period during which the respective dialysate was collected. * *p* < 0.05, ** *p* < 0.01, and *** *p* < 0.001 with respect to dialysate three (before antagonist injection), two-way ANOVA following by Tukey, *n* = 6 rats.

Drugs were diluted and perfused in the microdialysis media or intraperitoneal injected as indicated. In a set of experiments, the first three 20 min dialysates were collected to evaluate basal release, and then a perfusion of D3R antagonist GR 103,691 (100 nM) was initiated such that it was perfused into SNr from dialysate four to six and then continued sampling three more fractions (Figure 2) for a total of ten 20 min dialysates. In a second type of experiments (Figure 3, Figure 4 and Figure 5), after the collection of three basal dialysates, a first intra-nigral perfusion of antagonists (D1R antagonist SCH 23390: 1 µM, Figure 3; or NMDA receptor antagonist kynurenic acid: 500 µM, Figure 4; or a mixture of both, Figure 5) was performed and three more fractions were collected. After, an intra-nigral perfusion of GR 103,691 was conducted during three more dialysates and six more fractions were collected to make thirteen fractions in total. In the experiments of Figure 6, after collection of three basal dialysates, an intraperitoneal injection of the D3R antagonist U 99,194 was performed and six more dialysates were collected. Finally, in the experiments of Figure 7, before the administration of intraperitoneal U 99194A during collection of dialysate six, in dialysate three, D1Rs were blocked by perfusion of SCH 23,390 and maintained until dialysate nine. During all experiments, the automated locomotor box monitored the activity of rats. For each neurotransmitter evaluated, data were expressed as the percent of neurotransmitter measured in dialysate X with respect to the basal measurement of dialysate one. From the software of the automated locomotor recording system, ambulatory distance (cm), circling (turns/min), and non-ambulatory activity (counts) from each rat were obtained and graphed with respect to dialysate number. All data were analyzed by two-way analysis if variance (ANOVA) followed by the post hoc Tukey test, performed using GraphPad Prism (version 7.0). For comparison of the effects of drugs in dialysates from a different set of experiments, an unpaired *t*-test was performed. For description of the results, we highlight and indicate the statistical differences during the maximal effects of drugs; in graphs, main statistical differences are noted.

### 2.4. Drugs

Kynurenic acid, 4-Hydroxyquinoline-2-carboxylic acid, SCH 23,390, and R(+)-7-Chloro-8-hydroxy-3-methyl-1-phenyl-2,3,4,5-tetrahydro-1H-3-benzazepine hydrochloride were purchased from Sigma-Aldrich (St. Louis, MO, USA). GR 103,691, 4′-Acetyl-*N*-[4-[4-(2-methoxyphenyl)-1-piperazinyl] butyl]-[1, 1′-biphenyl]-4-carboxamide, U 99194A, 2,3-Dihydro-5,6-dimethoxy-*N*, and *N*-dipropyl-1H-inden-2-amine maleate were obtained from Tocris Biosence (Mineapolis, MN, USA).

## 3. Results 

### 3.1. D3R Blockade Increases Glutamate, Dopamine, and GABA in the SNr of Freely Moving Rats 

In order to test the role of D3R in modulating the release of glutamate, dopamine, and GABA in vivo, we first perfused into the SNr the highly selective antagonist GR 103,691 (100 nM i.c. [7]) in freely moving rats. The results of microdialysis coupled to behavior after the blockade of D3R are depicted in Figure 2. As can be observed, perfusion of GR 103,691 produced a substantial increment of glutamate from dialysate four to six, which returned gradually to the control value at dialysate seven (Figure 2A: glutamate maximal increase during dialysate number four: 239% ± 21% vs. dialysate number three: 102% ± 3%, mean difference −138, *p* < 0.001, F(9,63) = 35.5, two-way analysis of variance (ANOVA) followed by Tukey, *n* = 8 rats). In a similar manner, increases in dopamine and GABA with similar time courses to glutamate were found (Figure 2B,C, dopamine maximal increase dialysate number four: 202% ± 8% vs. dialysate number three: 99% ± 2%, mean difference −103, *p* < 0.001, F(9,63) = 81.46; GABA maximal increase dialysate number four: 170% ± 5% vs. dialysate number three: 100% ± 2%, mean difference −70, *p* < 0.001, F(9,63) = 54.28, and two-way ANOVA followed by Tukey, *n* = 8 rats). The simultaneous measurement of two parameters of locomotor activity (ambulatory distance and non-ambulatory counts) and one of locomotor asymmetry (turns/min) exhibited increases in the three parameters, which correlated with increments in glutamate, GABA, and dopamine levels with a similar time course (Figure 2D–F, maximal ambulatory distance during dialysate number four: 209 ± 20 cm vs. dialysate number three: 23 ± 13 cm, mean difference −186, *p* < 0.001, F(9,63) = 17.5; maximal non-ambulatory counts in dialysate number 4: 982 ± 35 counts vs. dialysate number 3: 250 ± 30 counts, mean difference −732, *p* < 0.001; F(9,63) = 23.02; maximal contralateral turns/min in dialysate number 4: 1.25 ± 0.25 turns/min vs. dialysate number 3: 0.12 ± 0.12, mean difference −1.25, *p* < 0.001, F(10,70) = 7.31, two-way ANOVA followed by Tukey, *n* = 8 rats).

### 3.2. D1R-Dependent Effects of D3R Blockade on GABA and Glutamate Release 

Previously, Rosales et al. [35,39], in similar microdialysis experiments in rat SNr, found that stimulation of the subthalamic nucleus leads to increases in GABA and glutamate levels. Those increases were mediated by D1R stimulation in GABAergic and glutamatergic terminals by dopamine, which was released by the glutamate, activating the NMDA receptors (NMDARs) in dopaminergic dendrites. Thus, we tested whether the increments in GABA and glutamate observed during D3R blockade are a consequence of D1R stimulation by endogenous dopamine released by glutamate. In a different set of rats, we perfused SCH 23390 (1 µM), a selective D1-like dopamine receptor antagonist [40], during dialysate four to nine, initiated prior to the blockade of D3R with GR 103,691 during dialysates seven to nine (Figure 3, red circles). For comparison, a set of six rats was perfused with saline instead of SCH 23,390 and with GR 103,691 in dialysate seven to nine (Figure 3, black circles). In this second set of animals, the effect of D3R blockade was not different from that observed in the experiments presented in Figure 2, in which the antagonist was perfused from dialysate four to six, in that statistical comparison of corresponding dialysates did not demonstrate significant differences (not shown). 

With respect to animals in which D1Rs were blocked, it can be observed (Figure 3A–C) that SCH 23,390 itself did not modify glutamate nor dopamine nor GABA baseline levels, even after 60 perfusion min (Figure 3A, glutamate dialysate number three: 98 ± 1 vs. dialysate number six: 100 ± 2, mean difference 2, ns, F(12,60) = 29.43; Figure 3B, dopamine dialysate number three: 99% ± 1% vs. dialysate number six: 101% ± 2%, mean difference −2, ns, F(12,60) = 50; Figure 3C, GABA dialysate number three: 99 ± 1 vs. dialysate number six: 96 ± 4, mean difference 3, ns, F(12,60) = 11.69; two-way ANOVA followed by Tukey (*n* = six rats)). Nevertheless, the D1R blockade produces diverse effects on the release of the neurotransmitters when D3R are also blocked. A decrease of the percentage of glutamate released by the D3R blockade was observed during the combined D1R blockade (Figure 3A, glutamate maximal increase dialysate number seven with GR 103,691: 244% ± 17% vs. dialysate number seven with GR 103,691 and SCH 23390: 153% ± 5%, mean difference 90, *p* < 0.001, F(12,240) = 147.6, two-way ANOVA followed by Tukey, *n* = 6 vs. 6 rats. Dopamine release was not modified by treatment (Figure 3B, dopamine maximal increase dialysate number seven with GR 103,691: 194% ± 6% vs. dialysate number seven with GR 103,691 and SCH 23390: 184% ± 8%, mean difference 10, ns, F(12, 240) = 80, two-way ANOVA followed by Tukey *n* = 6 vs. 6 rats). Finally, GABA release was completely abolished (Figure 3C, GABA maximal increase dialysate number seven with GR 103,691: 172% ± 6% vs. dialysate number seven with GR 103,691 and SCH 23390: 101% ± 3%, mean difference 70, *p* < 0.001, F(12,240) = 14.9, two-way ANOVA followed by Tukey, *n* = 6 vs. 6 rats). The most surprising finding is that blockade of D1R prevented the behavioral effects previously observed during D3R blockade alone (Figure 3D–F, red circles compared with black ones).

### 3.3. Increments in Glutamate and GABA Release by D3R Blockade Depend on Endogenous Dopamine

The results depicted in Figure 3 indicate that stimulation of D1R mediates part of the effect of D3R blockade on glutamate and the entire effect on GABA release. Previous reports have shown that glutamate controls dopamine release through activation of NMDAR [39,41,42]; therefore, we tested whether endogenous dopamine released through NMDAR activation by glutamate activates these D1Rs. The effects of the blockade of NMDAR with kynurenic acid (500 µM; [43,44]) during D3R blockade and during D3R plus D1R blockades were tested. Our results revealed that blockade of NMDAR did not modify basal glutamate release (Figure 4A, glutamate dialysate number three: 103% ± 4% vs. dialysate number six: 109% ± 4%, mean difference 6, ns, *p* = 0.99, F(12,60) = 33.7, two-way ANOVA following Tukey, *n* = six rats). However, the effect of D3R blockade on glutamate release remained (Figure 4A, glutamate dialysate number 6: 109% ± 4% vs. dialysate number 7: 172% ± 5%, mean difference −63, *p* < 0.001, F(12,60) = 33.68, two-way ANOVA followed by Tukey, *n* = 6 rats). Nevertheless, the effect is also less than that observed when D3R alone was blocked (see Figure 3A; mean glutamate dialysate seven: GR 103,691 244 ± 17 vs. Figure 4A kynurenic acised 172 ± 4, mean difference −71, F = 13.85, degrees of freedom (df) 5, *p* = 0.01, *n* = 6 vs. 6 rats, unpaired Student’s *t*-test), and nearly equals that observed with D1 and D3R blockade (see Figure 3A; mean glutamate fraction seven: GR 103,691 + SCH 23,390 154 ± 5 vs. Figure 4A kynurenic acid 172 ± 4, mean difference −18, F = 1.18, df 5, ns, *n* = 6 vs. 6 rats, unpaired Student’s *t*-test). On the other hand, as expected, perfusion with kynurenic acid prevented a dopamine increase mediated by D3R blockade (Figure 4B, dopamine dialysate number six kynurenic acid: 98% ± 4% vs. dialysate number seven: 100% ± 3%, mean difference 2, ns, F(12,60) = 0.27; two way-ANOVA followed by Tukey, *n* = 6 rats) without affecting baseline release (Figure 4B, dopamine dialysate number three kynurenic acid: 102% ± 2% vs. dialysate number six: 98% ± 3%, mean difference 4.5, ns, F(12,60) = 0.27; two way-ANOVA followed by Tukey, *n* = 6 rats). With respect to GABA levels, blockade of NMDAR prevented increments mediated by D3R blockade (Figure 4C, GABA dialysate number seven: 95% ± 1% vs. dialysate number seven of Figure 3C, 172% ± 6.13%, mean difference −77, F = 20.17, df 5, *p* = 0.005, *n* = 6 vs. 6 rats, unpaired Student’s *t*-test). Kynurenic acid did not modify baseline GABA release (Figure 4C, GABA dialysate number three kynurenic acid: 100% ± 3% vs. dialysate number six: 99% ± 3%, mean difference 1, ns, F(12,60) = 11.7; two way-ANOVA followed by Tukey, *n* = 6 rats). In addition, as observed with blockade of D1R, blockade of NMDAR also prevented behavioral effects of GR 103,691 (Figure 4A–C).

Finally, blockade of D1R and NMDAR concomitantly with administration of GR 103,691 was also tested. As can be observed in Figure 5, the effect is equal to that of the blockade of NMDA alone in the three neurotransmitters (Figure 4). An increase in glutamate levels was found, but it was lower than that observed with D3R blockade alone (Figure 5A, glutamate dialysate number six: 107% ± 3% vs. dialysate number seven: 171% ± 4%, mean difference −64, *p* < 0.001, F(12,60) = 44.48, two way-ANOVA followed by Tukey, *n* = 6 rats; comparison with Figure 3A; mean glutamate dialysate four: GR 103,691 244 ± 17 vs. Figure 5A kynurenic acid + SCH 23,390 171 ± 4, mean difference −73, F = 14.7, df 5, *p* = 0.01, *n* = 6 vs. 6 rats, unpaired Student’s *t*-test, *n* = 6 rats). In addition, no changes in dopamine level were observed (Figure 5B, dopamine dialysate number six, 103% ± 3% vs. dialysate number seven: 100% ± 4%, mean difference 2, ns, F(12,60) = 0.70; two way-ANOVA followed by Tukey, *n* = 6 rats), nor were there changes in GABA levels (Figure 5C, GABA dialysate number six, 102% ± 2% vs. dialysate number seven: 97% ± 4%, mean difference 5, ns, F(12,60) = 14.13; two way-ANOVA following Tukey, *n* = 6 rats). Finally, as observed with blockade of NMDAR alone, concomitant blockade with D1R prevented all behavioral effects of GR 103,691 (Figure 5A–C).

### 3.4. Systemic Blockade of D3R Mimics the Effect of Intra-Nigral D3R Blockade Role of Nigral D1R 

In order to determine whether the changes produced by D3R blockade in rat SNr correlate with changes induced by the systemic modification of D3R activity by the antagonist, we performed microdialysis experiments coupled to behavior in rats challenged by D3R antagonist U 99194A [2] administered intraperitoneally (i.p.). We decided to use U 99194A instead of GR 103,691 because the latter possesses less ability to cross the blood brain barrier [7]. The results of a single intraperitoneal injection of U 99194A (25 mg/kg i.p.) are displayed in Figure 6. Surprisingly, U 99194A induced a substantial increase in nigral glutamate (Figure 6A, glutamate maximal increase dialysate number four: 284% ± 19% vs. basal dialysate number three: 101% ± 2%, mean difference −182, *p* < 0.001, F(9, 45) = 34, two-way ANOVA followed by Tukey, *n* = 6 rats), which returned to baseline values at dialysate eight. In addition, increments in dopamine and GABA were observed during D3R systemic blockade (Figure 6B,C; dopamine maximal increase dialysate number four: 245% ± 13% vs. baseline dialysate number three: 101% ± 3%, mean difference −143, *p* < 0.001, F(9,45) = 75; GABA maximal increase dialysate number four: 239% ± 12% vs. baseline dialysate number three: 103% ± 3%, mean difference −136, *p* < 0.001, F(9,45) = 56.28, two-way ANOVA followed by Tukey, *n* = 6). As expected, U 99194A increased ambulatory distance and non-ambulatory counts, with a similar time course (Figure 2D,E, maximal ambulatory distance in dialysate number four: 474 ± 69 cm vs. baseline ambulatory distance during dialysate number three: 61 ± 28 cm, mean difference −413, *p* < 0.001, F(9,45) = 21.14; maximal non-ambulatory counts in dialysate number four: 1529 ± 347 counts vs. baseline non-ambulatory counts in dialysate number three: 372 ± 43 counts, mean difference −1157, *p* < 0.001, F(9,45) = 9.47; two-way ANOVA followed by Tukey, *n* = 6).

As nigral D1R blockade prevented the behavioral effects of the intra-nigral D3R antagonist, we tested a possible intra-nigral mechanism in systemic D3R blockade. Therefore, we conducted experiments blocking nigral D1R by the perfusion of SCH 23,390 from dialysate four to nine prior to the systemic injection of U 991194A during dialysate six; the results are depicted in Figure 7. As previously observed (Figure 3 and Figure 5), SCH 23,390 did not modify baseline glutamate, dopamine, or GABA (Figure 7A, glutamate dialysate number three: 97 ± 4 vs. dialysate number six: 98 ± 5, mean difference 1, ns, F(12,48) = 9.05; Figure 3B, dopamine dialysate number three: 98% ± 3% vs. dialysate number six: 99% ± 4%, mean difference −1 ns, F(12,60) = 6.63; Figure 3C, GABA dialysate number three: 98 ± 1 vs. dialysate number six: 105 ± 5, mean difference 6, ns, F(12,60) = 10.86; two-way ANOVA following Tukey, *n* = 6 rats). Nevertheless, intra-nigral blockade of D1R substantially decreased the increments in glutamate, dopamine, and GABA produced by the systemic administration of U 991,994 (Figure 6). For glutamate, a significant increment was observed in dialysate eight and nine (Figure 7A, glutamate dialysate three 98% ± 3% vs. dialysate eight 133% ± 6% and nine 142% ± 7%, mean differences −35 and −44, *p* < 0.001, F(12,60) = 16.33; two-way ANOVA followed by Tukey, *n* = 6 rats). In the case of dopamine, increments were also observed in dialysates eight and nine (Figure 7B, dopamine dialysate three 97% ± 2% vs. dialysate eight 124% ± 11% and nine 123% ± 4%, mean differences −27 and −27, *p* < 0.05, F(12,48) = 6.63; two-way ANOVA followed by Tukey, *n* = 6 rats). GABA increments were the highest of the three transmitters in dialysates seven and eight (Figure 7C, GABA dialysate three 98% ± 1% vs. dialysate seven 142% ± 10% and eight 156% ± 14%, mean differences −44 and −58, *p* < 0.001, F(12,60) = 21.12; two-way ANOVA followed by Tukey, *n* = 6 rats). Such changes in neurotransmitter produced low significant changes in ambulatory distance and non-ambulatory counts (Figure 7D, ambulatory distance dialysate three 43 ± 19 cm vs. dialysate eight 152 ± 26 cm and dialysate nine 191 ± 23 cm, mean differences −109 and −148, *p* < 0.01 and *p* < 0.05, respectively, F(12,48) = 6.9; non-ambulatory activity dialysate three 200 ± 49 counts vs. dialysate seven 590 ± 71 and dialysate eight 414 ± 93, mean differences −389 and −213, *** *p* < 0.001 and * *p* < 0.05, respectively, two-way ANOVA followed by Tukey, *n* = 6 rats).

**Figure 8:**
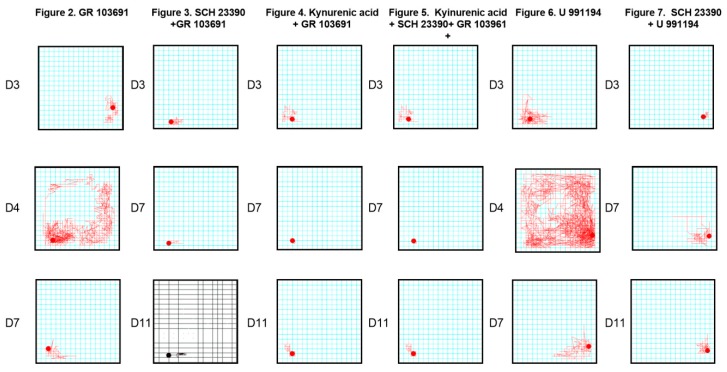
Graphic representation of the locomotor activity records of one rat of each experimental group performed by the automatized software of the locomotor activity box under the different experimental conditions explored in this study. The square represents the field area, and the red lines indicate the runs executed by animals. At the top is the figure that corresponds to the set of experiments, and on the left side of the figure is the corresponding 20 min dialysate.

In Figure 8, the graphic representation can be observed of the locomotor activity records of one rat of each experimental group performed by the automatized software of the experimental conditions explored in this study. It can be observed that the intra-nigral blockade of D3R produced an increase in locomotor activity (Figure 2 in the records), as well as systemic blockade (Figure 6 in the records), but that blockade of nigral D1R prevented or decreased the increments of locomotor activity produced by both manipulations of D3R blockade (Figure 3 and Figure 7 in the records).

## 4. Discussion 

Our data indicate that subthalamo-nigral presynaptic D3R, by control of glutamate release, modulates an interaction among glutamate–dopamine–GABA within the SNr. They also showed that an increment in the dopaminergic tone that glutamate exerts via NMDAR could not only modify interstitial glutamate itself, but that could also importantly modulate GABA release by D1R activation, consequently producing increments in motor activity. In addition, systemic blockade of D3R modifies neurotransmitter release in the same way as local blockade; thus, the intra-nigral effect could largely explain the behavioral effect of the systemic administration of D3R antagonists on motor behavior, revealing the pivotal role of subthalamo–nigral D3R on locomotor activity.

### 4.1. Considerations of Drugs Employed

To block intra-nigral D3R, the antagonist GR 103,691 was employed. This is a compound that possesses a higher affinity for D3R than for D2R, judged by its Ki = 0.4 nM [7] and EC50 = 6 nM for stimulation of glutamate release in nigral slices from not reserpinized rats (data not shown, for methods, see [26]. Two factors favor in that the antagonist at the concentration used almost completely blocked nigral D3R. First, on the basis of estimated barriers to drug diffusion in microdialysis reverse experiments [45], we used a concentration of 100 nM in our experiments to ensure activation of this type of receptor, reaching an approximate concentration estimated around 10 nM in the interstitial space. Second, the Ki of GR 103,691 for D2R (also present in subthalamo–nigral terminals) is 60 times higher than for D3R [7] and the relative contribution of D2R to the control of glutamate release is at least 75% less, compared with D3R within SNr [26]; thus, this ensured the preferential blockade of D3R, instead of D2R, by GR 103,691.

On the other hand, U 99194A is a preferential D3R antagonist that has a Ki ranging from 78 to 220 nM for D3R, which is 10–100 higher than for D2R [2,7] and an EC50 of 24 nM in nigral glutamate release (unpublished data). Moreover, U 99194A exhibits a weak antagonistic effect on D2R mediated responses, which is consistent with its low affinity for this type of receptor. Finally, U 99194A possesses higher permeation to the blood brain barrier and a better systemic distribution [2] than GR 103,691. Therefore, we decided to utilize U 99194A to block D3R by systemic administration in experiments of microdialysis and behavior motor.

### 4.2. Role of D3R at Dopamine, GABA, and Glutamate Afferents to SNr in the Neurochemical and Behavioral Effect of Intra-Nigral D3R Blockade 

As previously mentioned, SNr exhibits abundant expression of D3R in GABAergic and glutamatergic afferents, as well as in dopaminergic dendrites, but their roles in motor behavior are not completely understood. One would expect that the selective blockade or activation of D3R in one or more of these terminals or dendrites should produce changes in the interstitial levels of either glutamate, GABA, and/or dopamine. In response, such direct or indirect modification of neurotransmitters could modify the activity of nigro-thalamic neurons and, finally, motor behavior. In this work, we found that a unilateral microinjection of the highly selective D3R antagonist GR 103,961 [7] into the SNr produces an increase in general motor behavior and locomotor asymmetry (Figure 2D,E). From the latter, several questions arise. How does intra-nigral D3R blockade modify motor behavior? Are one or all D3Rs located in the nucleus responsible for the behavior observed? Let us first analyze, one by one, presynaptic D3Rs onto afferents for their possible role in explaining the neurotransmitter changes and motor behavior observed after D3R antagonist blockade. 

The first candidate to analyze, we thought, is the D3 autoreceptor, because it appears that the regulation of neurotransmitter release by the autoreceptor requires high affinity for their endogenous ligands and probably tonic activation in order to exert its function [46]. Activation or blockade of dendritic D3Rs, which regulate dopamine release by means of an autoreceptor function, could change locomotor activity through a modification of either GABA or glutamate release, in that afferents have presynaptic dopamine receptors. In this regard, dopamine controls glutamate release in subthalamo–nigral terminals mainly by highly sensitive D3R [23,25,26]. Thus, changes in interstitial dopamine could initially modify subthalamo–nigral D3R activity; thereby, D3 autoreceptor activation would decrease dopamine release and the activity of inhibitory subthalamo–nigral D3R. The latter would increase the glutamate level and could stimulate nigral output activity, which would inhibit thalamo-cortical neurons and decrease motor activity. Conversely, D3 autoreceptor blockade would increase dopamine, which would in turn stimulate subthalamo–nigral D3R, decreasing glutamate release and nigral output and increasing motor activity. This proposed mechanism is compatible with the effects observed on motor activity herein by D3R blockade (Figure 2D,E). However, such a mechanism implies tonic activity of D3 autoreceptors, which was not supported by our experimental data. Although our results demonstrated an increase in dopamine after D3R blockade (Figure 2B), the antagonism of NMDAR with kynurenic acid [43] (Figure 4B and Figure 5B) prevented the effect of this blockade and the level of dopamine remained equal to the baseline level, whereas the increase in glutamate was maintained (Figure 4A and Figure 5A). Thus, a tonic activity of the D3 autoreceptor could not be confirmed; however, a tonic effect of dopamine regulating glutamate release through presynaptic D3R (Figure 2A, Figure 3A, Figure 4A, and Figure 5A) was observed. These findings discard a substantial role of the D3 autoreceptor in the changes observed in neurotransmitter release and motor activity by D3R blockade. One possibility to explain the apparent lack of participation of a D3 autoreceptor on intra-nigral D3R blockade can be attributed to a previously suggested small role of D3 subtype autoreceptors on dopamine release [31,47]. 

The second explanation may be provided by the role of the striato–nigral D3R, whose function in controlling nigral GABA release depends on striato–nigral D1R activity [19,20]. However, under our experimental conditions, the striato–nigral D3R did not participate in the motor activity produced by D3R blockade; notwithstanding that D3R blockade also increased GABA release by D1R dopamine-mediated activation (Figure 2C), the potentiation produced by the D3R was blocked by the experimental manipulation and no additional effect of D1R on GABA release can occur.

The third candidate to explain the effects of D3R blockade on motor behavior is the subthalamo–nigral D3R, which is tonically activated by endogenous dopamine (Figure 2A, Figure 3A, Figure 4A, and Figure 5A). Subthalamic stimulation increases the activity of SNr neurons [48] in a complex mechanism that involves AMPA and NMDA receptor activation [49]. Thus, D3R blockade will produce an increase in glutamate release, which could lead to the activation of ionotropic receptors on GABAergic output neurons and the enhancement of their firing rate. In consequence, this will produce a decrease in thalamo-cortical activity, and thus decreased motor behavior. Nevertheless, our observations exhibited an opposite effect—an increase in motor activity despite the increase in glutamate release observed (Figure 2A), discarding a direct effect of glutamate on output neurons as being responsible for the behavioral effects observed. In the following section, we return to discussing the role of glutamatergic presynaptic D3R in this regard. In summary, neither the blockade of D3 autoreceptor nor presynaptic striato–nigral or subthalamo–nigral D3R and their possible effect on output neurons alone may explain the observed behavioral effects; thus, an alternative mechanism should explain the effect of D3R blockade on motor behavior.

### 4.3. Disruption of Reciprocal Interaction between Glutamate, Dopamine, and GABA as a Feasible Explanation of the Motor Effects of Intra-Nigral D3R Blockade

As the single blockade of one of the three intra-nigral D3R does not explain the behavioral effects, and because changes in the levels of the three neurotransmitters in microdialysis experiments were observed (Figure 2), it is possible that a more complex mechanism occurs in the nucleus to produce the increase in motor activity. That is why we explored the role of the reciprocal interaction among glutamate, dopamine, and GABA that we previously described [39]. In this interaction, stimulation of subthalamo–nigral neuron activity and glutamate released in SNr similarly increased the three neurotransmitters by a similar mechanism [39]. According to this proposal, changes in interstitial glutamate produce the stimulation of dopamine release via NMDAR and, subsequently, the dopamine released activates D1R located on striato–nigral and subthalamic afferents, reinforcing glutamate release and importantly promoting GABA release. The sequence of steps in this mechanism indicates that changes in interstitial glutamate are the first one [35]; in our experiments, we found that an increase in glutamate by the blockade of subthalamo–nigral presynaptic D3R also triggers these cascades of events. 

Tonic activation by dopamine of subthalamo–nigral D3R is present, in that it is the only neurotransmitter of the three that remains increased in terms of its baseline level after NMDAR (Figure 4 and Figure 5) and/or D1R (Figure 3 and Figure 5) blockade. Why were the other D3R not tonically activated by endogenous dopamine, but subthalamo–nigral was? According to our point of view, two factors could contribute; the first is the relatively high sensitivity of the subthalamo–nigral D3 presynaptic receptors [26]. The affinity for dopamine of subthalamo–nigral D3R, striato–nigral D3R, and autoreceptors has not been determined, but, from the comparison of the potency of a preferential D3R agonist, we are able to speculate [4]. The IC_50_ reported for PD 128,907 on inhibiting glutamate release is 7 nM [26], whereas the EC_50_ in striato–nigral D3R that potentiate D1R stimulation of GABA release is ≈70 nM [20], and for the inhibition of dopamine release in striatum, the IC_50_ is 250 nM [50]. These data suggest that the most sensitive of the three receptors might be the subthalamo–nigral receptors; thus, it is probable that low interstitial dopamine concentrations preferably activate these receptors. A second factor is the relative activity of subthalamo–nigral cells compared with dopaminergic and striato–nigral neurons. In vivo, subthalamic neurons exhibit intrinsic activity at firing rates ranging from 25 to 50 Hz [51], dopaminergic neurons of 5–10 Hz [52], and striato–nigral neurons are silent or slow: 2.1 Hz [53]. Under our experimental conditions, our baseline value of glutamate was 298 fmol/µL, compared with that of 5.82 of dopamine and 33.5 of GABA, reflecting the activity of their respective neurons. This indicates that the high activity of subthalamic neurons releases high levels of glutamate and requires a fine-tuning modulation that is provided by dopamine through D3R maintaining an intra-nigral glutamatergic tone. Thus, blockade of D3R by the injection of GR 103,691 disrupts this mechanism and triggers a cascade of activation of presynaptic and postsynaptic receptors, whose final effect promotes motor behavior. 

The second step in the increase of glutamate is the activation of NMDAR. Blockade of the tonic control of glutamate by D3R triggers an increase in interstitial dopamine by NMDA receptor activation (Figure 4B and Figure 5B), equal to that previously observed [35,39,54]. Although glutamate can modify dopamine release by AMPA receptor (AMPAR) and NMDAR [55], and both receptors are present in dopaminergic neurons [56], the latter is preferably activated by glutamate [56] (Figure 4B). In addition, this comprises a further stimulation of dopamine release over the baseline level (Figure 4B), because it was observed that the NMDA receptor antagonist does not modify the baseline dopamine level [57,58] (Figure 4B and Figure 5B). In addition, NMDAR activation in dopaminergic neurons modulates burst firing, which in turn is related to dopamine release [56,59,60], notwithstanding that the receptors are located dendritically [61]. 

Subsequently, the increased concentration of dopamine promotes the activation of D1R located at the striato–nigral and subthalamo–nigral afferents, because blockade of D1R with the highly selective antagonist SCH 23,390 [40] prevented the increase in GABA and decreased, but did not abolish, the interstitial glutamate (Figure 3A–C). Dopamine on presynaptic D1R in striato–nigral terminals has an EC_50_ of 584 nM [20] and in the subthalamo–nigral terminals, and EC_50_ of 1242 nM [26]. Likewise, the density of D1R in striatal afferents represents about 90% of total nigral D1R (subthalamic aferences probably possess the remaining 10%) [62]. Thus, increased dopamine initially activates preferentially presynaptic striato–nigral D1R, increasing interstitial GABA, inhibiting the activity of GABAergic projection neurons, and promoting motor behavior [33]. Single changes in dopamine and/or glutamate do not explain or contribute to explaining the motor behavior produced by intra-nigral D3R blockade: the GABA increment is necessary. A decrease in the firing rate of nigral output neurons that disinhibits thalamic premotor nuclei can produce stimulation of motor behavior, and this can be produced by the stimulation of striato–nigral presynaptic D1R [63,64,65] and GABA A post-synaptic receptors [66,67]. The fact that intra-nigral D1R blockade prevented the increment in GABA observed reinforces this proposal (Figure 3). This determinant role of presynaptic striato–nigral D1R and GABA A in the motor behavior observed is supported by several other facts as follows:

1. Mobilization of nigral GABA by intra-nigral D1R stimulation is sufficient to promote motor activity, despite that, most probably, both subthalamo–nigral and striato–nigral D1R are activated by the injection of agonist [63,64,65].
2. Blockade of D1R abolishes the increase in GABA level and motor behavior [66] (Figure 2). In addition, in our study, the blockade of NMDAR by kynurenic acid also prevented the GABA increment and motor activation (Figure 4 and Figure 5), indicating the lack of activation of striato–nigral D1R. No substantial tonic D1R activation exists under this experimental condition, in that SCH 23,390 did not significantly modify the baseline level of the three neurotransmitters.
3. Nigral blockade of GABA A receptors prevents all behavioral effects of the D1R agonist [67]. Thus, postsynaptic nigral glutamate receptors, despite their activation by released glutamate, appear not to participate directly in motor stimulation, but they do promote changes in GABA indirectly through the mobilization of dopamine by NMDAR activation.
4. GABA does not, but glutamate stimulates motor activity, because the intra-nigral GABA agonist muscimol induces contralateral turns, whereas the glutamate agonist induces ipsilateral circling [68,69].
5. Output neurons appear to possess more sensitivity to GABA than to glutamate with respect to changes in firing rate [51,70]. Additionally, nigral GABA A receptors are more densely expressed in nigra (GABA-sensitive binding: 382 fmol/mg protein [71]) than NMDA receptors (glutamate on NMDA receptors: 112 fmol/mg protein [72]).

However, it is possible that the activity of nigral NMDAR that stimulates the firing of SNc neurons may produce an increase in dopamine release, not only in SNr, but also in the striatum [73]. This might lead to the activation of D1R and D2R post-synaptic receptors located in striatal neurons, increasing/decreasing the activity of striato–nigral and striato–palidal neurons, respectively [74], thus contributing to the stimulation of the motor behavior observed. Nevertheless, the fact that the blockade of nigral D1R prevented all of the behavioral effects and abolished the increase in GABA release (Figure 3) precludes this possibility; thus, this stimulation of GABA release does not involve an external nigral mechanism. It is important to take into account that dopamine effects on striatal neurons are exerted upon activity stimulated by the cortex, and that this is in turn triggered by intention of movement [75]. Under our experimental condition, the animals do not feel like moving themselves, as can be assumed, because prior to D3R antagonist perfusion, the level of movement was very low (Figure 1A–C and Figure 8) and the animals were habituated for 1 h prior to experimental manipulation.

In a final step, dopamine activates presynaptic subthalamo–nigral D1R, increasing glutamate release; this glutamate reinforces the reciprocal interaction between neurotransmitters. How can this positive reciprocal interaction be stopped? Despite maintenance of the D3R blockade, neurotransmitter levels decrease and return to the baseline state; hence, there must be a factor regulating or stopping this mechanism. It is difficult to say what this factor is; however, local factors such as diffusion or metabolism could contribute. Nevertheless, some intra-nigral mechanisms could also contribute and one or more of the three neurotransmitters involved in the interaction could be this factor. For example, glutamate can decrease nigral GABA and glutamate release through presynaptic metabotropic receptors [76]; also, GABA_B_ receptor activation by released GABA could decrease the GABA release stimulated by D1R [77], and D1R and depolarization induced glutamate release [78] and the release of nigral dopamine [79]. Thus, by multiple regulation of the three neurotransmitters, GABA or glutamate receptors could regulate the effects of the increase in glutamate evoked by the blockade of the D3R, which is thus required in this regard. In conclusion, the blockade of intra-nigral D3R through an increase in glutamate triggers the release of dopamine and GABA, which stimulates the motor behavior. 

This type of local interaction among neurotransmitters awakened by D3R blockade requires spatial- and temporal-factor determinants for its operation. First, the structures involved should be in close proximity, so that the released neurotransmitters may activate the receptors involved in the interaction. Concerning this, Bevan et al. [80] employed anterograde tracers and demonstrated that subthalamic and striatal afferents converge onto the dendritic field of GABAergic output neurons. In addition, dopaminergic dendrites within the SNr receive afferents from the striatum and the subthalamus [81]; thus, anatomical distribution is suitable for interaction among neurotransmitters. This same anatomical disposition also permits direct actions of GABA onto GABAergic neurons.

**Figure 9:**
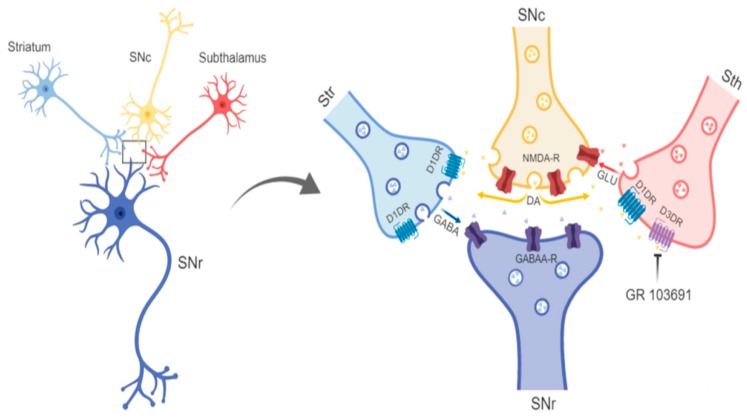
Drawing indicating intra-nigral local interaction among glutamate, dopamine, and GABA within the substantia nigra pars reticulata. The convergence of striatal, subthalamic, and nigra compacta dendrites on nigral output neurons is shown, as well as the presynaptic receptors participating in the interaction and the main site of the effect of the D3R antagonist. SNc, Substantia nigra pars compacta; Sth, subthalamic nucleus; DA, dopamine; GLU, glutamate.

Diffusibility of dopamine and glutamate can also be necessary, as some receptors, such as D1R and probably D3R and NMDAR, are extrasynaptic [82,83,84]. Diffusibility is favored by the spillover of dopamine [85] and glutamate, as suggested by the existence of *boutons en-passage* of subthalamo–nigral axons [86]. GABA A appears to be the only synaptic receptor involved in the interaction, although the immunolabelling of the GABA A extrasynaptic receptor has been reported [87,88]. Therefore, interaction between glutamate, dopamine, and GABA mediated by D3R is feasible. Figure 9 presents a drawing indicating the receptors involved in the interaction and the site of the main effect of the D3R antagonist.

### 4.4. Behavioral Effects of the Blockade of Nigral versus Systemic D3R

The most interesting result in this study is that systemic D3R blockade mimics the neurochemical and behavioral effects of the intra-nigral D3R blockade. During systemic D3R blockade, the nigral mechanism that we found by means of the local blockade appears to be a factor that amplifies the response, a mechanism in which presynaptic subthalamo–nigral presynaptic D3R and striato–nigral D1R play a pivotal role. 

From studies of systemic D3R blockade or stimulation with selective agonists, two hypotheses have been proposed to explain their effects on motor behavior. One proposal assumed that manipulations of dopamine D3 autoreceptor function are responsible for the behavioral effects [1,4,6,89]. The other one proposes that postsynaptic D3R, instead of presynaptic, D3 autoreceptors, are responsible for the observed effect [2,3,13,90]. This is supported by the fact that behavioral changes after the administration of D3R modifiers do not correlate with changes in the parameters of autoreceptor activity, such as dopamine concentration in the dopaminergic target nucleus or changes in dopamine synthesis [2,3,90,91]. 

From the second hypothesis, ventral striatum post-synaptic D3Rs are most probably the responsible candidates, because this region possesses one of the highest expressions [92], and local manipulations by microinjections also mimic the behavioral effects of systemic manipulations [93,94]. Our data indicate that there is also a significant role of nigral D3R, as the blockade of nigral D1R (Figure 3, Figure 5 and Figure 7) decreased the rises in nigral transmitters and motor activity elicited by systemic administration of the D3R antagonist. It is feasible to suppose that glutamate–dopamine–GABA interaction in the SNr represents an amplification mechanism of an effect also elicited in the ventral striatum, the consequence of this effect observed after nigral D1R blockade (Figure 7). 

The pivotal role of nigral D1R in motor behavior elicited by systemic manipulations is supported by previous observations. It was shown, in rats trained into a reinforcing conditioned motor behavior and in measurements of open-field locomotion, which blockade of nigral D1R is more potent in inhibiting the elicited behavior than accumbal or striatal receptors [66]. In another study, intra-nigral D1R blockade importantly diminished the motor effects induced by systemic amphetamines [95]. In addition, this main role of nigral D1R has also been demonstrated in motor pathological conditions, such as Parkinson’s disease [96,97], in which a nigral mechanism amplifies the motor effects of dopamine that can be initiated in outside areas. Importantly, the significant role of D1R in the effects of D3R blockade is also supported by the fact that the systemic blockade of D1R prevents the behavioral hyperactivity elicited by the D3R blockade [11,90] and that SNr is the most sensitive site for D1R blockade [66]. 

Finally, during intra-nigral D1R blockade, the most significantly incremented neurotransmitter was GABA, which could explain the increment in motor activity observed (Figure 7C,D). SNr also represents the output station of the ventral- and dorsal-ganglia baseline pathways [98]. Thus, it is feasible to think that nigral and accumbal mechanisms can occur simultaneously, because there are GABAergic projections to SNr that could be modulated by D3R [99,100,101].

## 5. Conclusions

In summary, a reciprocal interaction between glutamate, dopamine, and GABA triggered by subthalamo–nigral D3R blockade that involves activation of NMDA and D1R explains the motor behavior elicited by local D3R manipulations. This intra-nigral mechanism is important for the motor behavior modulated by D3R, and explains in part the effect of systemic pharmacological manipulations of D3R on motor activity.
